# Half brain irradiation in a murine model of breast cancer brain metastasis: magnetic resonance imaging and histological assessments of dose-response

**DOI:** 10.1186/s13014-018-1028-8

**Published:** 2018-06-01

**Authors:** Niloufar Zarghami, Donna H. Murrell, Michael D. Jensen, Frederick A. Dick, Ann F. Chambers, Paula J. Foster, Eugene Wong

**Affiliations:** 1 Department of Medical Biophysics, University of Western Ontario, London, Ontario Canada; 2 Department of Biochemistry, University of Western Ontario, London, Ontario Canada; 3 London Regional Cancer Program, University of Western Ontario, London, Ontario Canada; 4 Imaging Research Laboratories, Robarts Research Institute, London, Ontario Canada; 5 Department of Oncology, University of Western Ontario, London, Ontario Canada; 6 Department of Physics and Astronomy, University of Western Ontario, London, Ontario Canada

**Keywords:** Breast cancer, Brain metastases, Small animal radiation therapy, Radiation dose-response, Magnetic resonance imaging, DNA double-strand breaks, γ-H2AX

## Abstract

**Background:**

Brain metastasis is becoming increasingly prevalent in breast cancer due to improved extra-cranial disease control. With emerging availability of modern image-guided radiation platforms, mouse models of brain metastases and small animal magnetic resonance imaging (MRI), we examined brain metastases’ responses from radiotherapy in the pre-clinical setting. In this study, we employed half brain irradiation to reduce inter-subject variability in metastases dose-response evaluations.

**Methods:**

Half brain irradiation was performed on a micro-CT/RT system in a human breast cancer (MDA-MB-231-BR) brain metastasis mouse model. Radiation induced DNA double stranded breaks in tumors and normal mouse brain tissue were quantified using γ-H2AX immunohistochemistry at 30 min (acute) and 11 days (longitudinal) after half-brain treatment for doses of 8, 16 and 24 Gy. In addition, tumor responses were assessed volumetrically with in-vivo longitudinal MRI and histologically for tumor cell density and nuclear size.

**Results:**

In the acute setting, γ-H2AX staining in tumors saturated at higher doses while normal mouse brain tissue continued to increase linearly in the phosphorylation of H2AX. While γ-H2AX fluorescence intensities returned to the background level in the brain 11 days after treatment, the residual γ-H2AX phosphorylation in the radiated tumors remained elevated compared to un-irradiated contralateral tumors. With radiation, MRI-derived relative tumor growth was significantly reduced compared to the un-irradiated side. While there was no difference in MRI tumor volume growth between 16 and 24 Gy, there was a significant reduction in tumor cell density from histology with increasing dose. In the longitudinal study, nuclear size in the residual tumor cells increased significantly as the radiation dose was increased.

**Conclusions:**

Radiation damages to the DNAs in the normal brain parenchyma are resolved over time, but remain unrepaired in the treated tumors. Furthermore, there is a radiation dose response in nuclear size of surviving tumor cells. Increase in nuclear size together with unrepaired DNA damage indicated that the surviving tumor cells post radiation had continued to progress in the cell cycle with DNA replication, but failed cytokinesis. Half brain irradiation provides efficient evaluation of dose-response for cancer cell lines, a pre-requisite to perform experiments to understand radio-resistance in brain metastases.

## Background

The parallel developments of modern image-guided preclinical radiotherapy devices, small animal magnetic resonance imaging, and mouse model of brain metastasis presents us with a unique opportunity to ask brain metastasis-specific radiobiology questions. We, and others, have recently employed whole brain irradiation in mouse models of brain metastasis due to breast cancer to study tumor response after different timing or fractionation regimens of radiotherapy [1–3]. Despite the use of a tumor bearing animal model, inter-subject variability remained the major contributor to experimental uncertainties requiring typically 6-12 animals per longitudinal study group each lasting approximately 30 days, making these studies challenging.

Examples of contributors to inter-subject variability include variations in the number of cells delivered to the brain from intra-cardiac injection, number of proliferating metastases, and their subsequent growth [4]. In addition, post-sacrifice immunohistochemistry (IHC) slide staining results can also vary despite following the same protocol [5]. This led us [6] and others [7] to develop and validate platforms for specifically half-brain irradiations [8], allowing us to reduce inter-animal and inter-histological slide variability by using the contralateral brain as the control.

Due to these challenges, tumor radiation dose-response is generally not well established in-vivo, and we expect that the dose-response would depend on cell lines and sublines with specific genes inserted or deleted. In this study, we present our dose-response findings from our half brain irradiation of the brain metastasis mouse model using a well published human triple negative cell line MDA-MB-231-BR. Endpoints include both tumor metastases volumes from longitudinal magnetic resonance imaging brain imaging and histological endpoints.

Ionizing radiation induced DNA double-strand breaks (DSBs) are known to be lethal lesions that are responsible for cell’s mitotic death [9]. In response to DSBs, a histone H2A family member X, H2AX, is rapidly phosphorylated to form γ-H2AX [10]. Staining for γ-H2AX are therefore be employed as a measurement of DNA DSBs [11]. It is known that tumors have higher amounts of “cryptogenic” γ-H2AX due to endogenous sources such as replication stress, genomic instability, uncapped telomeres and apoptosis compared to the healthy tissue [12–14]. Previous studies have investigated the residual γ-H2AX of murine normal tissues from days to two months after exposure to detect radiation-induced toxicity such as fibrosis and myelopathy [15–17]. To the best of our knowledge, tumors’ residual γ-H2AX after in-vivo irradiation has not been previously reported.

The aim of this study is to measure the radiation dose-response of a breast cancer brain metastases model to radiation using half-brain irradiation to reduce inter-subject variability. We accomplished this using two animal cohorts. In the first cohort, DNA DSBs within cancer cells and the brain was assessed via immunohistochemistry staining of γ-H2AX in the acute setting (30 min after half-brain treatment) at three radiation dose levels. Tumor dose-response over time was evaluated in the second cohort using longitudinal MRI (prior to and 11 days after half-brain treatment) as well as immunohistochemistry at the endpoint using two radiation dose levels. MRI was used to obtain tumor volumes. In addition to assessing DNA DSB, 4′,6-diamidino-2-phenylindole (DAPI) immunohistochemistry staining of the cell nuclei was used to assess tumor cell density and nuclear size. By performing half brain irradiations in conjunction with MRI and immunohistochemistry in the acute and longitudinal settings, we were able to compare responses in the tumors versus normal mouse brain tissues, and radiated tumors versus un-irradiated tumors in the same animal at the various dose levels.

## Methods

Table 1 provides an overview of the study experiments performed and analyzed. We will describe them in more details in this section.

**Table 1:** Summary of experiment: number of animals and MRI-identified irradiated metastases for the acute and longitudinal study

Study	Group	Number of Mice	Minimum number of tumors visualized on MR		Dose (Gy)	Dissection after radiation therapy
			Irradiated	Shielded		
Acute (dissection after 30 min)	A	3	90	90	8	30 minutes
	B	3	90	90	16	30 minutes
	C	4	120	120	24	30 minutes
			Number of tumors tracked on longitudinal MR			
			Irradiated	Shielded		
Longitudinal (dissection 11 days)	A	3	68	85	16	11 days
	B	3	49	60	24	11 days

### Cell culture

For this study, the brain tropic clone of human triple-negative breast cancer cell line, MDA-MB-231-BR, stably transfected with enhanced green fluorescent protein (EGFP) was used [18]. Cells were cultured and maintained in Dulbecco’s modified Eagle’s medium (DMEM) containing 10% fetal bovine serum and 1% penicillin/streptomycin. Cultured cells were kept in 5% CO_2_ at 37 °C. Trypan blue exclusion assay was done to determine cell viability.

### Animal tumor model

To deliver MDA-MB-231-BR cells into the brain, the intra-cardiac injection method was used to distribute cells through arterial circulation. Female nu/nu mice (*N* = 19, 6–8 weeks old; Charles River Laboratories) were anesthetized with 1.5 to 2% vaporized inhaled isoflurane in O_2_. A suspension containing 1.5 × 10^5^ MDA-MB-231-BR cells in 0.1 ml of Hanks balanced salt solution was slowly injected into the left ventricle of the beating heart of the mouse [19]. Animals were housed in ventilated cages with a 12-h light/dark cycle and controlled temperature (20-22 °C), fed normal chow and given water ad libitum. Animal’s appearance and behavior was scored daily through the experiment and no profound effect of pain and distress on behavior was observed. This study followed animal care protocols approved by the Animal Use Subcommittee of The University of Western Ontario and were consistent with the policies of the Canadian Council on Animal Care. Mice received half brain radiation 26 days after cell injection.

### Mouse half-brain irradiation

**Fig. 1:**
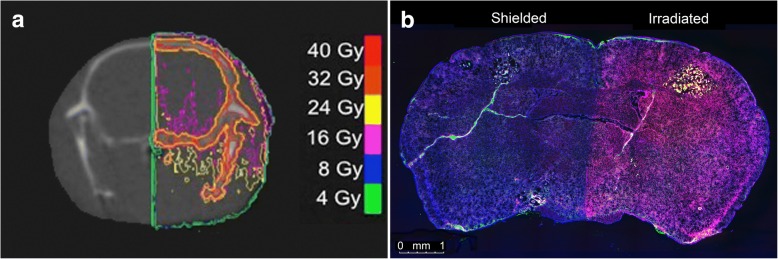
**a** Calculated dose distribution on coronal CT plane of the mouse brain for a 16 Gy (magenta isodose line) half brain irradiation. **b** Whole brain image of γ-H2AX stained section (red), imaged at 10X. DAPI counterstaining of DNA is shown in blue. Stable EGFP labeled tumors are in green. γ-H2AX stain shows the sharp edge of the beam in the middle of the brain along the longitudinal fissure

Mice received half brain radiation therapy on the modified GE eXplore CT 120 (GE Healthcare, Milwaukee, WI) preclinical imaging system [20, 21]. They were anesthetized using 1.5 to 2% vaporized inhaled isoflurane and were immobilized using the customized 3D-printed mouse head holder with a targeting accuracy of < 0.15 mm [6]. Mice were set-up in a feet first prone position. The longitudinal fissure (LF) was visually set as the anatomical target for the radiation field. Setup lasers and CT images were used to verify the alignment of the animal’s head in the head holder. Once the mouse was immobilized for treatment, online dorsal-ventral fluoroscopy was acquired to identify the rim of the skull and to position the collimators. A small CT localization marker was placed on the right side of the head holder to help with animal orientation on CT and fluoroscopy. The right half of the brain was irradiated with a single field (14 × 20 mm^2^) from the dorsal direction. Mice received doses of 8, 16 or 24 Gy in a single fraction. These dose levels were chosen because the biological effective dose (BED, assuming α/β = 10 Gy) of 16 Gy and 24 Gy in a single fraction are meant to represent doses prescribed for whole brain radiation therapy (30 Gy in 10 fractions) [22, 23] and stereotactic radiosurgery respectively (18-24 Gy in one fraction) [24]. Figure 1 shows a representative dose distribution in the mouse brain for 16 Gy. The 16 Gy iso-dose line (magenta color) in Fig. 1 shows homogenous radiation dose for the hemisphere away from the field edge near the midline of the brain. We have measured the dose drop off to be 7.5% per 5 mm [20]. We prescribed the dose to the midplane of the brain, and expected then the variation to be +/− 3.75%. That is, when we prescribed 16 Gy to the midplane, the variation across the brain will be 16 Gy +/− 0.6 Gy. This dose variation is minimal compared to the dose levels of 8, 16 and 24 Gy. The dose received by the un-irradiated side of the brain and tumors were denoted as 0* and will be employed as the control of the irradiated side in the same mouse. After recovery from radiotherapy, mice were selected either for acute or longitudinal dose-response study.

### In-vivo MRI

All mice were imaged on a 3 T GE clinical MR scanner (General Electric, Mississauga, Canada) with a custom-built gradient insert coil on day 26 after tumor injection and before receiving radiation. MRI was performed to verify the presence of the tumors in the mouse brain, particularly in both brain hemispheres. Mice that had no identifiable brain metastases on MR did not proceed to RT and excluded from this study. Images were acquired using 3D balanced steady-state free precession (bSSFP) protocol (acquisition resolution = 100 × 100 × 200 μm, repetition time = 8 ms, echo time = 4 ms, flip angle = 35°, receive bandwidth = 19.23 kHz, signal averages = 2, radiofrequency phase cycles = 8, scan time = 29 min, along with ZIP2 and ZIP512 upscaling), a well-established imaging technique for this model [25–27]. To evaluate the response of breast cancer brain metastases to different radiation doses in-vivo, the longitudinal group was imaged again 11 days after receiving half brain radiotherapy (37 days after tumor injection) with the same imaging protocol.

### MRI analysis

**Fig. 2:**
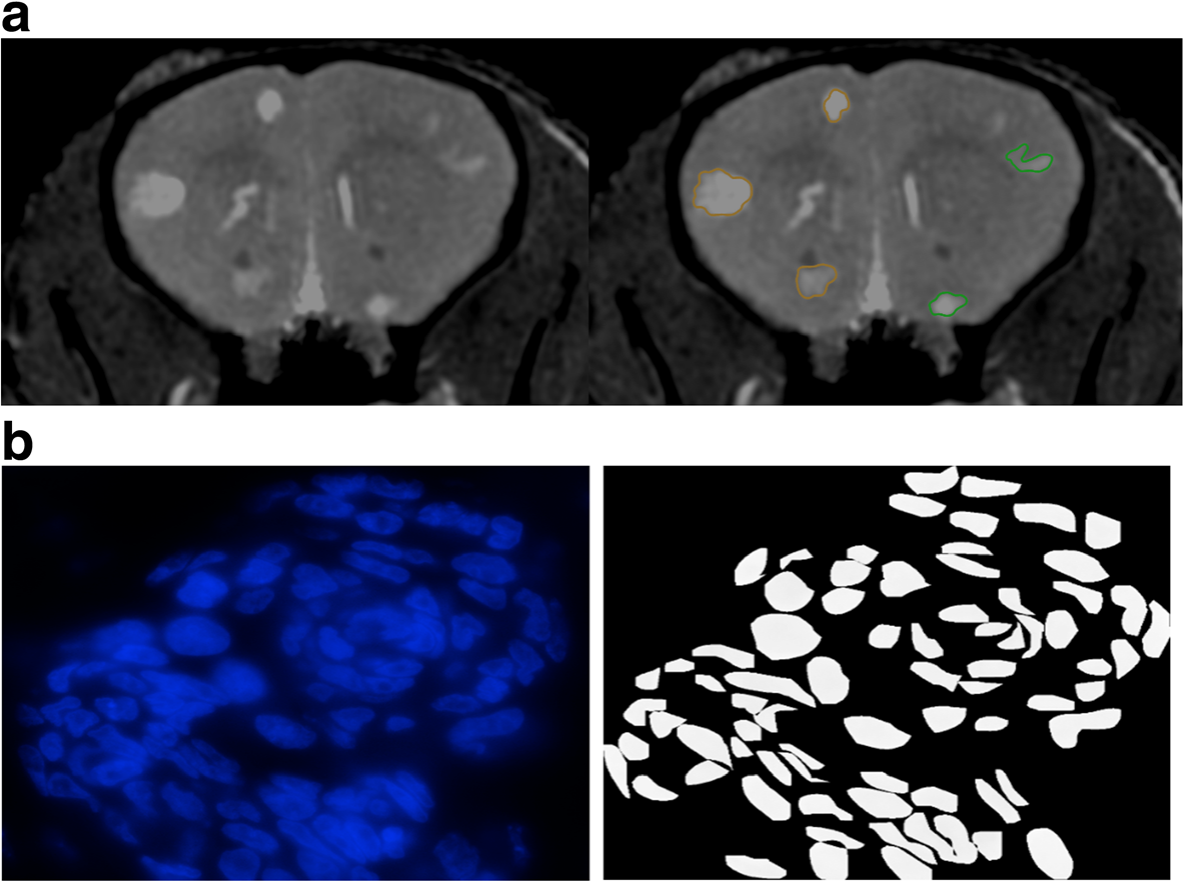
**a** Manual segmentation of tumors on an MR scan acquired 11 days after RT of an animal treated to 24 Gy to the right brain. Original MR image is on the left panel and segmented MR image is on the right. Tumors segmented in green are in the right (irradiated) half of the brain, and tumors segmented in orange are in the left (shielded) brain. **b** An example of our segmentation of DAPI-stained tumor nuclei. Original DAPI image of a tumor cluster is shown on the left panel. Segmented tumor nuclei are shown on the right which we employed in our analyses

Brain metastases were segmented manually on pre and post-radiotherapy images by a single observer using open-source OsiriX image software version 6.0. Tumors in the midline of the brain (±200 μm of the longitudinal fissure) were excluded from the study as only part of these tumors may have been irradiated. Figure 2a showed an example of the manual segmentation of the tumors performed on an MR acquired on day 11 after RT. Mean fractional volume changes of the tumors were calculated by dividing the post-treatment tumor volume by the volume of the same tumor before treatment and averaged for all brain metastasis for mice in each group. One mouse in the 24 Gy longitudinal cohort had to be sacrificed at 7 days due to its deteriorating condition.

### Immunohistochemistry

At the two post-irradiation time-points (30 min or 11 days) mouse brain samples were collected and processed for immunohistochemistry staining. Mice were perfused with 0.9% saline followed by 4% paraformaldehyde (PFA). Brains were harvested and post-fixed in 4% PFA and transferred to 30% sucrose solution until the specimen sank to the bottom. Brain samples were embedded in Tissue-Tek OCT Compound (Sakura, Torrance, CA) and frozen. Cyrosectioning of coronal slices was performed with 10-μm slice thickness. Tissue sections were stained with hematoxylin and eosin (H&E) to assess the morphology of the tumors.

Immunostaining was performed with the primary monoclonal antibody against γ-H2AX using a protocol published by Ford et al. [28]. Staining of sections consisted of antigen retrieval with sodium citrate, 1 h incubation in blocking serum (10% goat serum with 0.1% Triton X-100 for membrane permeabilization), overnight incubation at 4 °C in mouse anti-γ-H2AX antibody (anti-phospho-histone H2AX, Ser139, clone JBW301; Millipore, Billerica, MA, USA) at the dilution of 1:700, 1 h incubation in secondary antibody (1:500 goat anti-mouse Alexa Fluor 594 conjugated, Life Technologies, Carlsbad, CA, USA.), DAPI counterstain 5 min, and mount with anti-fade mounting medium Vectashield (Vector Laboratories, Inc. Burlington, ON). This protocol was used consistently to stain sections from the two time-points. For quantification, images were acquired with 100X (oil immersion) objective lens on a fluorescence microscope (Carl Zeiss Canada Ltd). Imaging parameters such as intensity, exposure time and gain were kept consistent during the experiment. We collected a total of ten to thirteen images of different tumors for each mouse.

### Histological quantification

To evaluate the DNA damage response, γ-H2AX stained sections of tumors were analyzed for each radiation dose level. The amount of damage was also quantified in neighboring normal brain tissues under the same conditions as the tumors. Initially, we employed an inverted confocal microscope (Olympus Fluoview FV1000 Confocal Imaging System) for high resolution 3D images of γ-H2AX foci within the nuclei [29]. We observed in the acute setting γ-H2AX foci were over-lapping, which made detection of individual foci impossible. Similarly, foci saturation was observed in the irradiated tumors in the longitudinal experiment. Unable to count individual foci, we quantified γ-H2AX based on the fluorescent stain intensity, which is a more reliable method for high radiation doses [30, 31].

All IHC analyses were performed on images taken from the fluorescence microscope using 100X oil immersion objective. The γ-H2AX intensity was measured for both normal mouse brain and tumor tissues. Tumor nuclei were visually distinguished from mouse nuclei based on the characteristic punctuate pattern of mouse DAPI staining [32]. To quantify γ-H2AX intensity, DAPI-stained nuclei were used to generate nuclear outlines in which the γ-H2AX intensity would be measured. Nuclear segmentations were used to eliminate signal from background fluorescence. Nuclei on DAPI images were manually segmented using Adobe Photoshop CC. For each field of view, total γ-H2AX fluorescence intensity was obtained by summing the intensity values of all pixels within the segmented boundary using an in-house code developed and validated in MATLAB (MathWorks, Natick, MA, USA). The total γ-H2AX fluorescence intensity for each field of view was normalized to the total area of segmented nuclei for the same field (Eq. 1).

1 $$ \kern2em \upgamma -\mathrm{H}2\mathrm{AX}\ \mathrm{intensity}\ \mathrm{density}=\frac{\mathrm{Total}\ \upgamma -\mathrm{H}2\mathrm{AX}\ \mathrm{intensity}\ \mathrm{in}\ \mathrm{segmented}\ \mathrm{nuclei}}{\mathrm{Total}\ \mathrm{area}\ \mathrm{of}\ \mathrm{segmented}\ \mathrm{nuclei}} $$

Mean γ-H2AX intensity per unit area was determined for each treatment condition in the acute and longitudinal settings. The total number of nuclei analyzed for each dose level varied from 350 to 950.

We observed that MDA-MB-231-BR tumors grew in clusters surrounded by edema. We obtained the number of tumor nuclei per cluster area. This index gave us the density of tumor nuclei/cells in each cluster (Eq. 2).

2 $$ \mathrm{Tumor}\ \mathrm{cell}\ \mathrm{density}=\frac{\mathrm{Number}\ \mathrm{of}\ \mathrm{tumor}\ \mathrm{nuclei}\ \mathrm{in}\ \mathrm{cluster}}{\mathrm{Area}\ \mathrm{of}\ \mathrm{segmented}\ \mathrm{cluster}} $$

**Fig. 3:**
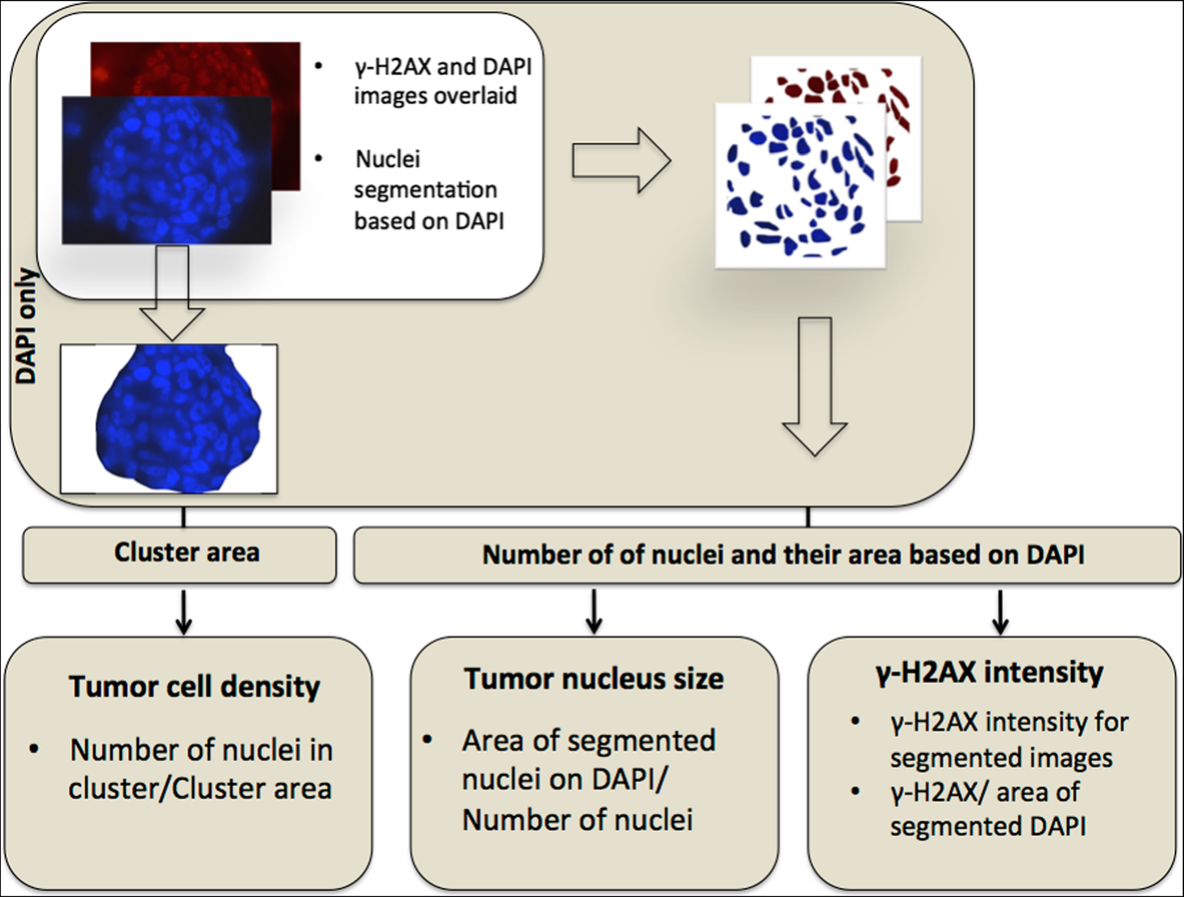
Flow chart of the processes involved in the quantification of γ-H2AX intensity, tumor nucleus size and tumor cell density. DAPI and γ-H2AX images were overlaid and nuclei were segmented based on DAPI. The intensity of γ-H2AX from segmented nuclei was acquired. From the segmented DAPI images, number and total area of segmented nuclei were quantified. For tumor cell density analysis, tumor clusters were segmented based on DAPI and the area of the cluster was computed

We quantified both the tumor cell density and size of tumor nucleus for all radiation doses at the two time-points. Figure 3 shows the flow chart of the processes involved in these histological quantifications. IHC staining was repeated three times for the acute study and twice for the longitudinal study.

We also observed an increase in tumor nuclei size and we quantified the size of tumor nuclei by computing the average area of each nucleus from DAPI images (Eq. 3).

3 $$ \mathrm{Average}\ \mathrm{area}\ \mathrm{of}\ \mathrm{tumor}\ \mathrm{nucleus}=\frac{\mathrm{Total}\ \mathrm{area}\ \mathrm{of}\ \mathrm{segmented}\ \mathrm{nuclei}}{\mathrm{Number}\ \mathrm{of}\ \mathrm{segmented}\ \mathrm{nuclei}} $$

### Statistics

Statistical analyses were performed using SPSS (Armonk, NY: IBM Corp) and confirmed by GraphPad Prism software (La Jolla, CA, USA). The normality of the measured variables was tested using the Shapiro-Wilk test and the *p* < 0.05 was used as the significance threshold. For normally distributed variables, between-groups analysis of variance (ANOVA) followed by Tukey *post-hoc* test was conducted to determine whether the response was statistically significant (*p* < 0.05). Nonparametric Kruskal-Wallis analysis followed by Mann-Whitney U test was used for variables that were not normally distributed.

## Results

### γ-H2AX radiation dose-response

In the acute radiation dose-response study, mice received half brain radiation of 8, 16 and 24 Gy (minimum *N* = 3 per dose) and were sacrificed approximately 30 min after treatment. Tissue sections were stained for γ-H2AX to quantify the initial damage induced in both normal mouse brain and tumors. Figure 1b displays a mouse whole brain coronal section, which received half brain radiation of 16 Gy.

**Fig. 4:**
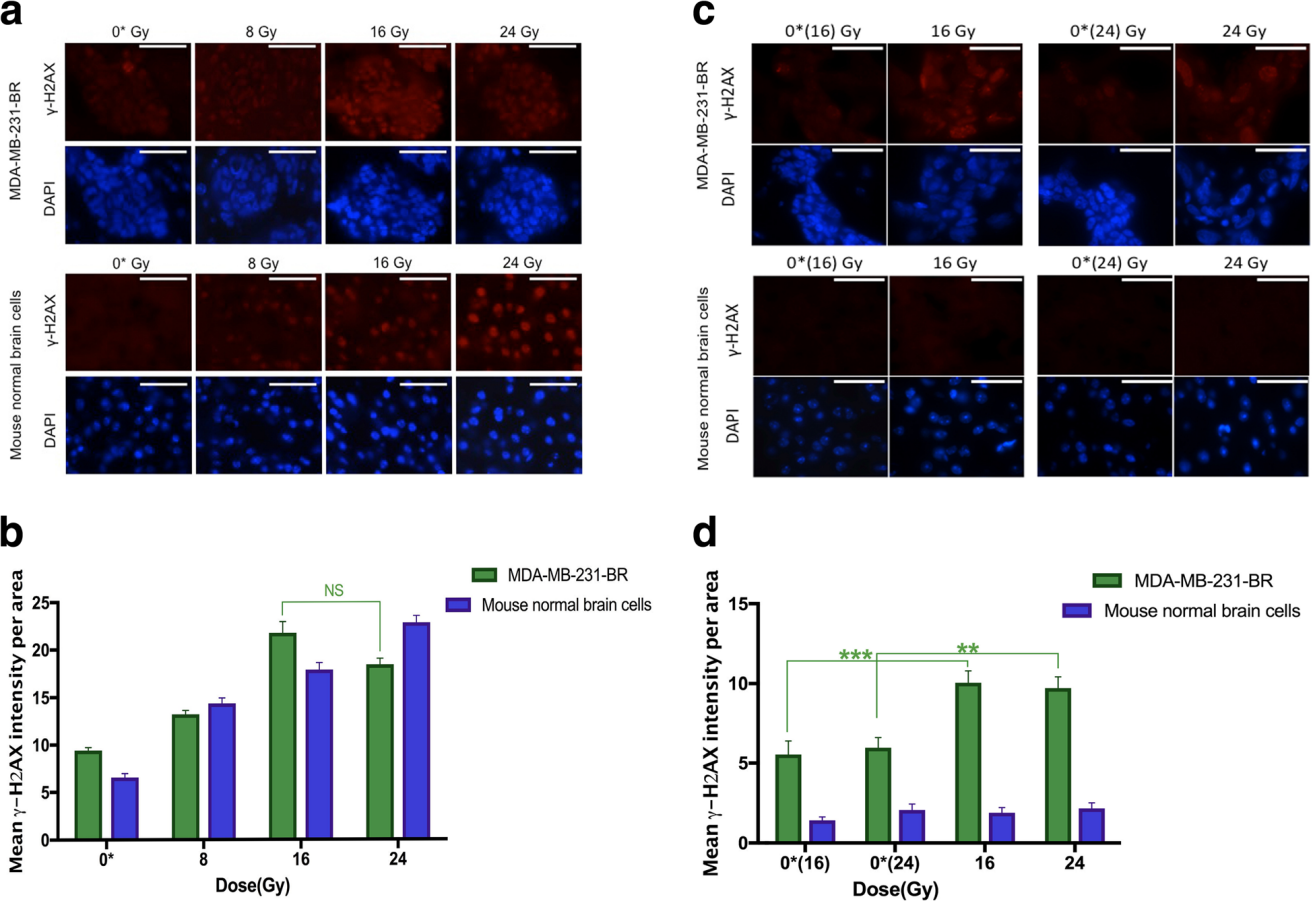
**a** Acute DNA damage response 30 min post-irradiation. Histology sections of fluorescent γ-H2AX and corresponding DAPI (nuclei) stained for tumor (MDA-MB-231-BR) and normal brain are shown. Images were taken with a fluorescence microscope (100X objective). Scale bar = 50 μm. **b** Quantification of the intensity of γ-H2AX staining versus radiation dose 30 min after radiotherapy. Tumors are plotted in green and normal brain tissue are plotted in blue. In irradiated normal brain tissue, the γ-H2AX intensity had a linear trend (R^2^ = 0.78, *p* < 0.001). In tumors, γ-H2AX did not continue to increase at the dose of 24 Gy even though the γ-H2AX intensity is significantly different between irradiated and un-irradiated sides (*p* < 0.0001). Error bar indicates standard error of the mean. **c** Residual DNA damage response 11 days post-irradiation. Scale bar = 50 μm. (**d**) Quantification of the intensity of γ-H2AX staining for the various radiation dose 11 days after radiotherapy. In normal brain, γ-H2AX intensities returned to the background level. In irradiated tumors, γ-H2AX intensity was higher than both the background level and tumors in the irradiated side. ** = *p* ≤ 0.01, *** = *p* ≤ 0.001, and error bar indicates standard error of the mean

Figure 4a shows the tissue sections of tumors and normal mouse brain stained with DAPI and γ-H2AX at the acute time point. Figure 4b shows our quantification of γ-H2AX based on fluorescence intensity density in the nuclei of normal brain and tumor tissues evaluated at the acute time point. In normal brain, the amount of γ-H2AX intensity density increased linearly (R^2^ = 0.78, *p* < 0.001) with increasing radiation dose. However, in tumors, this trend stopped at 16 Gy; the level of γ-H2AX intensity density dropped at the dose of 24 Gy compared to 16 Gy. The γ-H2AX intensity density in both tumors and normal brain of the irradiated side were significantly increased (*p* < 0.0001) compared to the respective un-irradiated side (8 versus 0*(8), 16 versus 0*(16) and 24 versus 0*(24) Gy).

To investigate how much of the initial damage is retained in both tumors and normal brain tissues, γ-H2AX intensity density was measured for the longitudinal group 11 days after hemi brain radiation (Figs. 4c, d). We observed that γ-H2AX intensity density in irradiated normal brain nuclei returned to background levels when compared to un-irradiated side of the brain 11 days after radiotherapy. However, irradiated tumors had higher levels of γ-H2AX intensity density compared to tumors in the contralateral un-irradiated sides (0*(16) and 0*(24) Gy). There was no significant difference in the amount of residual γ-H2AX between irradiated tumors (16 Gy vs. 24 Gy).

### In-vivo dose-response

**Fig. 5:**
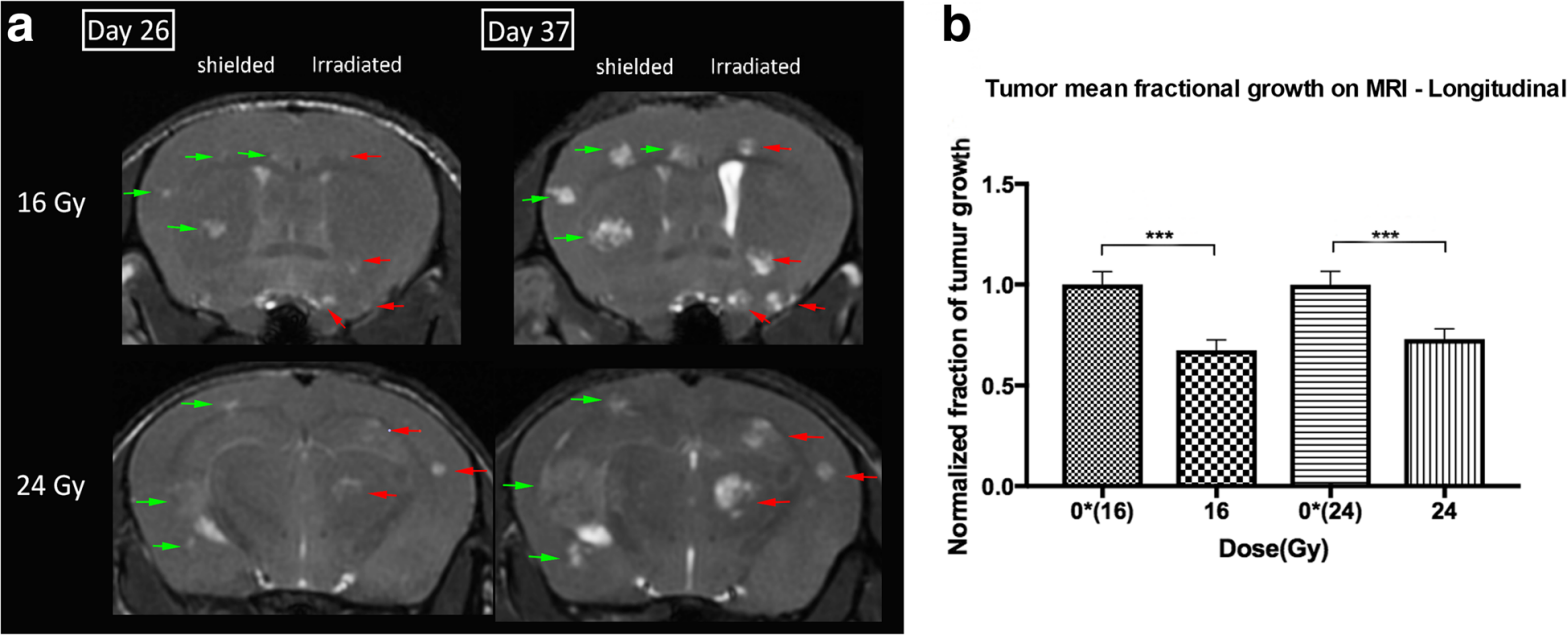
**a** MR images (bSSFP) of the mouse brain at two-time points. Metastases appear as hyper-intense (bright) regions compared to brain parenchyma. Pre-treatment images are on day 26 and images on day 37 are for the same mouse 11 days after radiation therapy. Right half of the brain was irradiated. One mouse per radiation group is shown. Red arrows indicate the brain metastases in the irradiated side while green arrows show brain metastases in the un-irradiated side. **b** Mean fractional growth of brain metastases measured on MR images for the radiation doses normalized to that of the un-irradiated halves. Tumors irradiated with 16 and 24 Gy grew with significantly different growth rates than their respective un-irradiated sides (Kruskal-Wallis followed by Mann-Whitney U test). No difference was observed between irradiated tumors of 16 and 24 Gy. *** = *p* ≤ 0.001, error bar indicates standard error of the mean

To assess the changes in the volume of tumors in response to radiation doses in-vivo, MR images were taken before and 11 days after half brain radiation therapy. Representative images of brain metastases at two different time-points for doses of 16 and 24 Gy are shown (Fig. 5a). The mean fractional growth of the tumors was calculated for each group (Fig. 5b). There was a statistically significant difference (Mann-Whitney U *p* **≤** 0.05) between the growth of un-irradiated and irradiated brain metastases for both doses of 16 and 24 Gy. A second observer segmented tumors on MRI on two animals treated at 24 Gy and confirmed this finding. The fractional reduction in tumor volume growth as assessed by MRI was not statistically different between 16 and 24 Gy in the longitudinal setting. Tumor Cell Density.

**Fig. 6:**
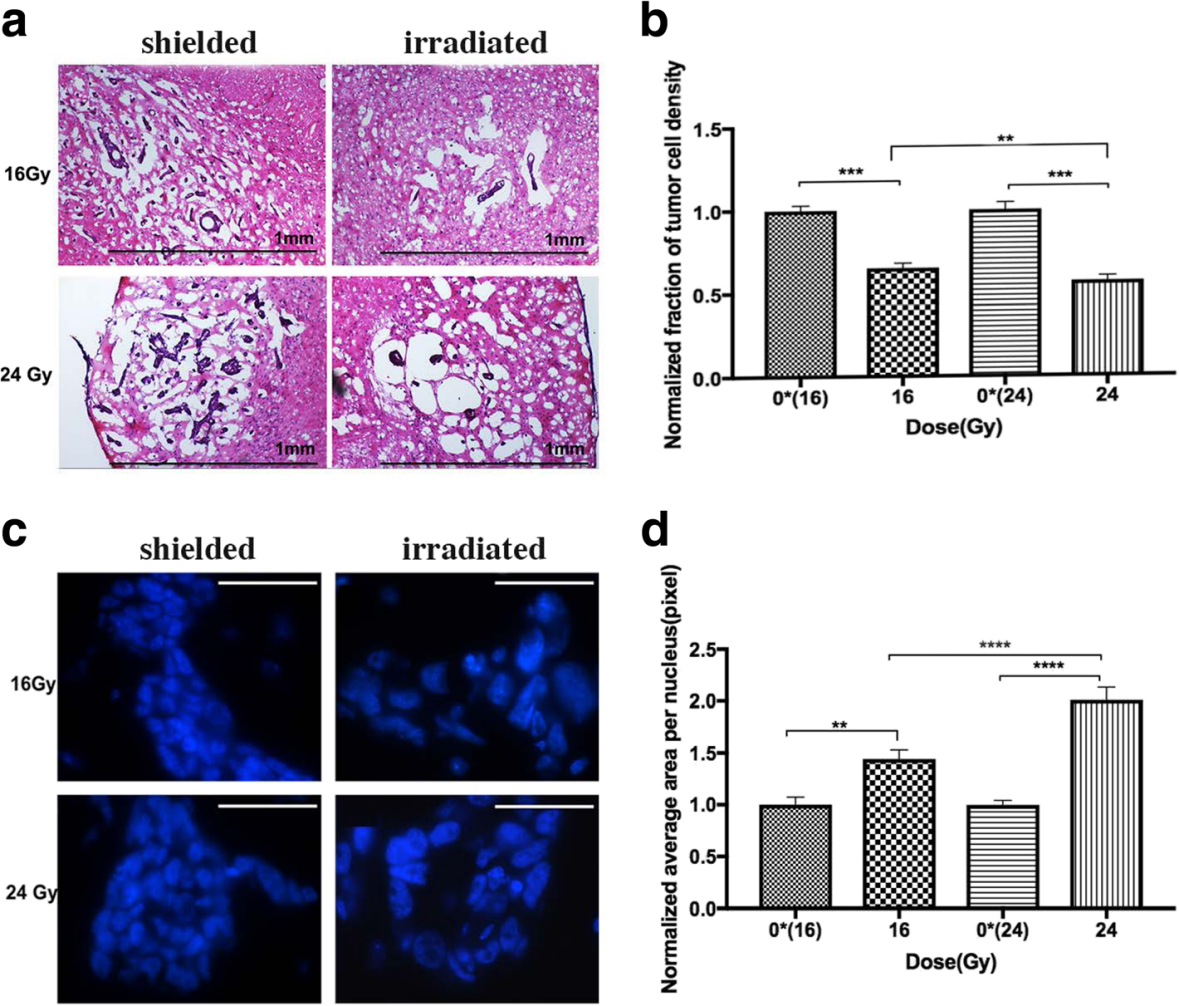
**a** H&E stained sections of shielded and irradiated tumors from the same section of a mouse brain 11 days after radiotherapy at 16 and 24 Gy (10X magnification). Scale bar = 1 mm. **b** Quantification of tumor cell density 11 days after radiotherapy. The densities of tumor cells treated with 16 and 24 Gy were significantly lower than their corresponding un-treated side. There was also a significant difference between treated tumors at 16 and 24 Gy. **c** DAPI staining of shielded and irradiated tumor nuclei from the same section of a mouse brain 11 days after radiotherapy at 16 and 24 Gy. Scale bar = 50 μm. **d** Average size of tumor nuclei 11 days after radiotherapy normalized by that of the respective un-irradiated halves. There was a significant difference between the sizes of tumor nuclei treated with 16 and 24 Gy compared to the contralateral side. The size of tumor nuclei was also significantly different between 16 and 24 Gy. ** = *p* ≤ 0.01, *** = *p* ≤ 0.001, **** = *p* ≤ 0.0001, error bar indicates standard error of mean

We observed on H&E samples from the longitudinal cohort that irradiated tumors are less compacted with cells, and surrounded by a more substantial amount of edema compared to tumors on the un-irradiated side (Fig. 6a). We quantified this by calculating tumor cell density based on DAPI staining for tumors in both the acute and longitudinal settings. The acute setting was employed to provide a baseline verification. As expected, no significant difference was detected in the density between treated and un-treated tumors and for different radiation doses 30 min after radiation.

On the other hand, there was a significant difference in tumor cell density between treated and un-treated tumors in the longitudinal experiment (Fig. 6b). Furthermore, there was a significantly lower density in those treated with 24 Gy compared to 16 Gy.

### Tumor cell nuclear size

DAPI is used as a counterstain for the nucleus of the cell and we used this stain to investigate the size of tumor nuclei for both acute and longitudinal studies. We observed that the nuclei of treated tumors were significantly larger than the un-treated nuclei 11 days after radiotherapy. Figure 6c shows the different morphological appearances of irradiated versus un-irradiated tumor nuclei stained with DAPI. The size of tumor nuclei was quantified for both acute and longitudinal studies. The acute setting quantification was employed to establish a baseline and no significant differences was found in the average size of tumor nuclei 30 min after treatment. A second observer repeated this DAPI nuclei segmentation on tumors that were treated at 24 Gy and their contralateral control and confirmed the manual segmentation results. However, in the longitudinal cohort, there was a significant difference in the size of the nuclei between treated and un-treated sides of the same mice. Radiation dose at 24 Gy resulted in a significantly larger nuclei size than 16 Gy in the longitudinal setting (Fig. 6d).

## Discussion

In this study, we used both in-vivo and ex-vivo methods to evaluate the response of MDA-MB-231-BR brain metastases and normal brain to different radiation doses at two time-points after treatment. In the longitudinal study, the normal brain’s response contrasted with the tumors’ after delivering 16 or 24 Gy half brain irradiation: γ-H2AX levels returned to normal in brain nuclei 11 days after radiation, while tumors retained significantly higher density of phosphorylated γ-H2AX compared to un-irradiated tumors. This higher amount of phosphorylated γ-H2AX is independent of the increase in the size of the tumor nuclei that we also observed because we have quantified γ-H2AX intensity per unit nuclei area. It has been shown that tumors that retain the induced γ-H2AX in the first 24 h after radiotherapy are more likely to die [33]. This is supported by our imaging finding that tumors in the half brain treated with radiotherapy had significantly slower growth than tumors in the untreated side. Higher cryptogenic level of γ-H2AX in tumor cells [14] is attributed to dysfunctional telomeres that drives genomic instability [34]. Sustained elevation of γ-H2AX here could be predictive of an unstable genome, and may allow the acquisition of more aggressive characteristics [35] if the higher level of residual DSBs do not keep these cells from going through mitosis. Smart et al. [3] have successfully retrieved the surviving tumor cells after radiotherapy using the same animal model, and showed that they are more radiosensitive than before. Our results are consistent with this finding as we showed that remaining tumor cells after radiation has a higher sustained level of DNA damage with an elevated γ-H2AX.

We found that the tumor nuclear size increased at 16 and 24 Gy compared to contralateral controls (Fig. 6 c, d). This suggests that while DNA replication had continued, cells failed to undergo cytokinesis. When cell division is not possible, this leads to aneuploidy, polyploidy [36], or multinucleated cells [37, 38]. Cancer cells are known to exhibit aneuploidy, and here, we showed radiation further exacerbate this problem in cells that survived radiation in a dose-dependent manner.

Finally, we evaluated the response of treated and un-treated breast cancer brain metastases with MRI. In the bSSFP sequence, MDA-MB-231-BR brain metastases appear as hyperintense regions compared to normal mouse brain due to tumor-associated edema [1, 39, 40]. We found that treated tumors grew significantly less over 11 days compared to control, but not in a dose dependent manner. In contrast, histology sections of these tumors showed tumor cell density decreased with increasing radiation dose. It is expected that higher doses will lead to increased cell kill, but edema must set in to achieve a lower tumor cell density. One interpretation is that there exists a dose-response relationship of radiation induced edema, particularly in this cell line, and such edema masked the tumor volume response as assessed by bSSFP MRI. Diffusion MRI has the ability to detect such changes in tumor cell density and should be employed for future studies.

This study was limited by the exponential tumor growth in the MDA-MB-231-BR model which left a short interval (maximum of about 11 days) between MRI-visible metastasis and the need to sacrifice. This left us with a limited opportunity to observe longer term changes in gross tumor volume beyond what we have reported. Moreover, while half brain irradiation allowed us to reduce inter-animal and inter-slide staining variability, this technique can potentially introduce radiation-induced bystander effect [41]. We assumed in this work that the bystander effect is small in this brain metastasis model due to the use of nude mice that lacked adapative immune T-cells.

## Conclusions

Brain metastasis is a growing problem in breast cancer patients and new treatment strategies for brain metastasis are necessary. Radiotherapy is an established treatment that is currently used to treat the majority of brain metastasis patients. Understanding the properties of cancer cells surviving radiotherapy can provide evidence for further improvements (e.g. molecularly targeted adjuvant therapies) and optimization in the clinics. As a first step toward this goal, we evaluated the radiation dose-response of MDA-MB-231-BR breast cancer brain metastases in the present study. We found in the acute setting that γ-H2AX in tumors, unlike normal tissues, become saturated at the higher dose levels. In the longitudinal setting 11 days after treatment, we showed that the response of irradiated tumors (at both 16 and 24 Gy) differed from un-irradiated counterparts in γ-H2AX fluorescence intensity, MRI-assessed tumor growth, tumor cell density, tumor cell nuclear size, and the fraction of tumor cell proliferation. Decreased tumor cell density and increased nuclear size were seen when we increased the dose from 16 to 24 Gy, but not in γ-H2AX intensities or MRI tumor volume. We conclude that surviving MDA-MB-231-BR cells in the irradiated tumors must have continued DNA replication but failed cyctokinesis in a dose-dependent manner, leading to increased nuclear size. Furthermore, lower tumor cell density implied the presence of radiation induced edema for this cell line. Additional pre-clinical research is warranted to further understand these responses, their generalizability, and ultimately to capitalize on such information to improve brain metastasis radiotherapy.

## Abbreviations

ANOVA: Analysis of variance
BED: Biological effective dose
bSSFP: Balanced steady-state free precession
DAPI: 4′,6-diamidino-2-phenylindole fluorescent nuclear stain
DMEM: Dulbecco’s modified Eagle’s medium
DSB: Double stranded break
EGFP: Enhanced green fluorescent protein
H&E: Hematoxylin and eosin
H2AX: H2A histone family, member X
IHC: Immunohistochemistry
LF: Longitudinal fissure
MRI: Magnetic Resonance Imaging
PFA: Paraformaldehyde
